# Phytochemical screening and antimicrobial activity of *Polygala sadebeckiana Gürke* extracts on bacterial isolates from Wound samples of patients with *“Shimetere”*

**DOI:** 10.1186/s12906-024-04371-y

**Published:** 2024-02-01

**Authors:** Bereket Zeleke, Zebene Mekonnen, Meskele Bireda, Melaku Yitbarek, Andamlak Dendir

**Affiliations:** 1 Department of Pharmacy, College of Medicine and Health Science, Wolkite University, Wolkite, Ethiopia; 2 Department of Nursing, College of Medicine and Health Science, Wolkite University, Wolkite, Ethiopia; 3 School of Medicine, College of Medicine and Health Science, Wolkite University, Wolkite, Ethiopia; 4 Department of Public Health, College of Medicine and Health Science, Wolkite University, Wolkite, Ethiopia

**Keywords:** Antibacterial activity, *Polygala Sadebeckiana*, Skin infection, Methanol crude extract, Phytochemical screening

## Abstract

**Background:**

Modern medicine is not the choice of patients with *“shimetere”* in the Gurage community owing to their perception of ‘parenteral medication use severely aggravates the disease’. For this reason, the root part of Polygala sadebeckiana Gürke is commonly utilized as traditional medicine in the management of the disease. The aim of this study was to evaluate the antimicrobial activity of Polygala sadebeckiana Gürke extract on bacterial isolates from wound samples of patients with “*Shimetere*”.

**Methods:**

*The agar well diffusion* method was used to evaluate antibacterial activity, and the *agar dilution method* was utilized to determine minimum inhibitory concentrations (MICs) and minimum bactericidal concentrations (MICs). The crude extract was tested against isolated bacteria at concentrations of 25, 50, 75 and 100 mg/mL in triplicate (3x). The positive controls were azithromycin (15 µg) and cloxacillin disk (5 µg), and the negative control was dimethylsulfoxide (5%). The group mean comparisons were made using one-way ANOVA at a significance level of *p* < 0.05, and the results are presented as the mean ± standard deviation. The presence of secondary metabolites from crude extract was checked by standard testing procedures.

**Results:**

*S. aureus* and *S. pyrogen* were the two identified bacteria from 9 (60%) and 3 (20%) wound samples, respectively. All identified bacterial strains were susceptible to the reference antibiotics. Tannins and saponins were the most abundant secondary metabolites found in the crude extracts. The average inhibition zones of the plant extracts with 100, 75, 50 and 25 mg/mL concentrations were 27, 20.33, 15.25, and 11.96 mm (*p* < *0.000*) for *S. aureus* and 30.02, 24.50, 19.07, and 15.77 mm (*p* < *0.000*) for *S. pyrogen* bacteria, respectively. The MIC and MBC of the crude extract were 1.67 and 10 mg/mL for *S. aureus* and 0.98 and 4 mg/mL for *S. pyrogen*.

**Conclusion:**

*Polygala sadebeckiana Gürke* contained significant tannins and saponins as secondary metabolites and had antibacterial activities against isolated bacteria (*S. aureus* and *S. pyrogen*) from “*Shimetere*”. The potential mechanism of antibacterial action of the plant extract was cell wall synthesis inhibition.

## Introduction

Human skin is thought to be the first-line defense by acting as a physical barrier that protects against microbial attack. However, when the defensive barrier is damaged, soft tissue and skin infections may develop. Cellulitis is the most common skin infection involving the dermis and subcutaneous tissues, and *Staphylococcus aureus (S. aureus)* is the main cause of these infections [1].

Bacterial soft-tissue infections and skin are among the major communal health concerns and are hard to treat owing to the high occurrence of resistant bacterial strains, such as methicillin-resistant *S. aureus (MRSA)*, to the oldest generation antimicrobials in addition to the unavailability and the higher cost of newer generation drugs [1, 2].

*“Shimetere”* (its direct Amharic meaning ‘‘simu yemaytera’’, English meaning “never call its name”), which is considered a common skin infection in the Gurage community. According to many clinicians working at the health care facilities of the Gurage Zone, *“shimetere” has similar* clinical presentations as cellulites. The symptoms of redness, pain, swelling and heat are the common characteristics of cellulitis and *“shimetere”*. However, modern medicine is not the choice of patients with *“shimetere”* in the community because the fear of medication use via the injection route might seriously aggravate the disease. For this reason, *Polygala sadebeckiana Gürke* is commonly utilized as a traditional medicine [3, 4].

**Fig. 1:**
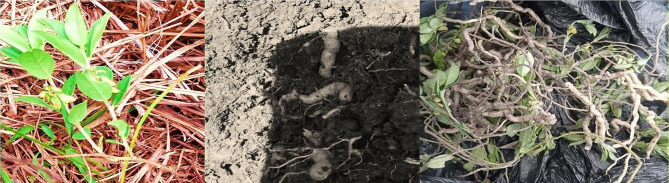
*Polygala sadebeckiana Gurke* plant and its root part in the Gurage zone

*Polygala sadebeckiana Gürke* belongs to a family of *Polygalaceae*. It is a perennial or annual herb up to 15(–30) cm high, vegetative parts puberulous with curved hairs and widely distributed in Ethiopia, Sudan, Kenya, Tanzania, Malawi and Mozambique. Locally, it is also known as *“yeshimetere chiza*, *felfel* or *qiteriye”.* It is commonly grown in low land areas of the Gurage zone, specifically Mihur-Aklil, Ezha and Cheza Woredas. A sample of the plant and its root part are shown below (Fig. 1). The root part of the plant is sold on the market and used orally or topically for medicinal use in the treatment of *“shimetere”* [2, 5].

Antimicrobial-resistant bacteria such as MRSA are associated with many clinical problems worldwide and are considered another concern for modern medicine use. The misuse of various antimicrobial drugs in health care facilities is considered the main reason behind the emergence of antimicrobial resistance. Antimicrobial overuse in health care facilities also has a significant role in the transmission of resistant microorganism strains [6–9].

Antimicrobial activity of traditionally used medicinal plants studies is also important in helpful locally accessible and significant plant species, especially for the detection of crude drugs, as herbal medicine might be harmless and would reduce resistance created by the pathogens, as they occur in a joined form of more than one molecule in the plant cell [10–13].

However, to date, no experimental study has been carried out on *Polygala sadebeckiana Gürke*, although the plant is claimed to have antimicrobial activity and is widely utilized as a medicinal plant to treat *“shimetere”* in the community. Therefore, the aim of this study was to evaluate the antimicrobial activity of *Polygala sadebeckiana Gürke* extract on bacterial isolates from wound samples of patients with *“shimetere”.*

## Methods and materials

### Description of plant collection areas

Plants were collected by botanists and traditional healers from selected districts of the Gurage Zone. The Gurage zone is located in southern central Ethiopia. It is situated at 37° 50′ 0″–38° 40′ 0″ E and 7° 40′ 0″–8° 30′ 0″ N. The Gurage zone covers an area of 5893.5 km^2^ with an altitude range of between 1000 and 3600 m a.s.l [5]. The mean yearly temperature is within the range of 13–30 °C and receives an annual rainfall ranging from 600 to 1600 mm. Based on the recent classification of vegetation types in Ethiopia, the study area is largely covered by dry evergreen Afromontane forest and grassland. A land use/cover map of the study area indicated that the cultivated land covered 52% and that only 9.9% was covered by natural and human-made forest [14]. This study was conducted from February 2022 - June 2023 GC.

*Polygala mooneyi M.G. Gilbert*, which is synonym with *Polygala sadebeckiana Gürke*, its specimen was formally collected and identified by M.G. Gilbert, Sebsebe D. and K. Volleson, #8055 from Sidamo (Southern Ethiopia) in 1986, and the specimen was deposited in the National Herbarium (ETH), with voucher ID number ETH000000016 [15].

### Study design

A qualitative phytochemical screening test and in vitro antibacterial evaluation were performed through a randomized experimental design. All experimental tests were performed three times (3×) together with the negative and positive controls.

### Plant collection and preparation

Fresh and healthy roots of *Polygala sadebeckiana* were identified and collected by botanists in collaboration with traditional medicine practitioners from different geographical districts of the Gurage Zone. Then, it was washed many times using sterilized distilled water, cut into smaller sizes of approximately 1–2 cm long, and dried under shade at room temperature for fifteen days. By using a wooden-made mortar and pestle, it was ground. Then, it was pounded into a fine powder using an electric grinder. Finally, it was kept in a refrigerator until use [16].

### Plant extraction

Twenty to 95% of solvent (polar or/and nonpolar) substances are frequently used by the herbal medicine industry to prepare crude plant extracts, although a standardized extraction protocol has not yet been developed. Previous studies indicate that methanol is a good solvent to extract bioactive chemicals from medicinal plants for antibacterial activities. The powdered root of the plant was macerated in 80% methanol in a conical flask for 2 days with occasional shaking. The filtrate was separated from the residue by Whatman No. 1 filter paper, and the residue was remacerated by additional methanol three to five times until the filtrate was free of any visible colour. The filtrates were combined and dried in an oven at a temperature of 40 °C to remove methanol water. The dried extract (percentage yield of 11.4%) was weighed and placed in a tightly closed amber-colored glass jar and kept in a refrigerator until use [16–18].

### Phytochemical analysis

Phytochemical tests were carried out for the methanol extract of the plant using standard procedures to identify the presence of secondary metabolites, including alkaloids, flavonoids, terpenoids, tannins, polyphenols, glycosides, phytosterols, and saponins [16].

#### Test for alkaloids

0.25 gm of the crude extract was added to five drops of HCl and then filtered, and finally, the filtrate was mixed with Wagner’s reagents. A brown precipitate formation indicated the presence of an alkaloid.

#### Test for

saponins: First, 0.25 gm of the crude extract was mixed with 20 mL of distilled water and shaken for 15 min in a graduated cylinder. The formation of foam in the 1 cm layer revealed the existence of saponins.

#### Test for polyphenol

Using the ferric chloride test, 0.25 gm of the crude extract was added to 4 drops of FeCl3. The formation of a blue‒black colour indicated the existence of phenols.

#### Test for flavonoids

Using an alkaline reagent test, a few drops of NaOH solution were added to the crude extracts. The formation of an intense yellow colour that became colourless with the addition of 10 drops of 1% HCl revealed the presence of flavonoids.

#### Tests for phytosterols

The Szarkowski test was performed by adding a few drops of chloroform to 0.25 gm of the crude extract and filtering. The filtrate was then mixed with some drops of concentrated H2SO4, shaken and left for a few minutes. The golden yellow color indicated the presence of phytosterols in the crude extract.

#### Test for tannins

0.25 gm of the crude extract was mixed with 1% gelatin solution containing NaCl. The formation of a white precipitate indicated the existence of tannins.

#### Tests for glycosides

The Keller-Kiliani test was employed. A total of 0.25 gm of plant extract was dissolved in 2 mL of glacial acetic acid containing 1 drop of FeCl3 solution. The mixture was poured into a test tube that contained 1 mL of concentrated H2SO4. A brown ring at the interphase indicated the presence of glycosides.

### Bacterial isolation, characterization and identification

Patients with “*Shimetere*” in the community, diagnosed by local traditional healers, were enrolled in the study. Samples were taken from the infection sites (wound surface) for bacterial isolation, characterization and identification. The wound surface was rinsed with sterile normal saline, and then samples were collected using sterile cotton swabs. The internal surface of the infected area was swabbed slightly. All swabs were immediately applied to freshly prepared MacConkey, blood and nutrient agar plates and then streaked and incubated overnight at 37 °C for 24 h. Following incubation, bacterial colonies were detected, and discrete colonies were picked. Afterwards, discrete colonies were refined by sub-culturing onto freshly prepared nutrients, MacConkey and blood agar using a streak plate technique. Isolated colonies that grew on the plates were transferred onto nutrient agar slants with an appropriate label. These agar slants were then stored in the refrigerator at 4 °C for further characterization. Characterization and identification of the bacterial isolates was based on standard microbiological methods, including morphological, gram staining and cultural characteristics on nutrient agar media, coagulase test, catalase test, oxidase test, indole production test, citrate test, triple sugar iron test and urease test [19].

### In vitro antibacterial activity test

Using the *Agar-Well Diffusion Method*, the identified microbial strains were utilized to evaluate the antibacterial activities of the crude plant extracts. *The broth dilution method* was used to determine the MIC and MBC of the crude plant extracts.

### Agar-well Diffusion Method

Following inoculation of identified bacterial strains with sterile swabs at the surface of Mueller Hinton agar plates, they were allowed to waterless at room temperature. Six holes were created at equal distances from each other on the Mueller Hinton agar plate surface. The holes were then filled with the crude plant extracts at different concentrations of 100, 75, 50 and 25 mg/mL, the negative control of 5% dimethyl sulfoxide (DMSO) and the positive control of antibiotic disks. The antibiotic disks were cloxacillin (5 µg/disk) for *S. aureus* and azithromycin (15 µg/disk) for *S. pylori bacterial isolates*. The experiment was repeated three times (3x). The plates were then kept at room temperature for approximately 1 h to favor diffusion and incubated at 37 °C for 24 h. After 24 h of incubation, the antibacterial activity was determined by measuring the inhibition zone (IZ) diameter including the hole. The result of bacterial inhibition was evaluated by comparison with growth in negative and positive controls. The susceptibility pattern of isolated bacteria to the reference drug was determined by measuring the IZ after 24 h of incubation [16, 20, 21].

### Broth-dilution method

*The* MIC and MBC of the crude extract were determined against *S. aureus* and *S. pyrogen by using the* broth dilution method with slight modifications [20, 21]. DMSO (5%) was used to dilute the crude extracts, and the concentration of plant extracts ranged from 0.5 to 50 mg/mL. After serial dilution, 0.1 mL of the extract was mixed with 0.1 mL of Mueller Hinton agar and poured into different test tubes. Fifty microliters of standardized inoculum (5 × 105 CFU/mL) was added to each test tube except the negative control and incubated at 37 °C for 24 h. Then, MIC was judged by comparison with growth in positive and negative controls. Finally, MBC was determined by incubation for the MIC test in 150 µL broth in the test tube and incubation for 48 h at 37 °C. The experiment was performed three times (3x) to confirm the data.

### Data analysis

The data were analysed by using Statistical Package for Social Science (SPSS) version 26 and are presented as the mean ± SD of three replicates. Statistical differences in the mean IZ for individual bacteria with differences in concentrations of the extract were analysed using one-way analysis of variance (ANOVA) followed by Tukey’s post hoc test at a significance level of *p* < 0.05. MIC and MBC were analysed using descriptive statistics. The phytochemical tests were recorded as + (plus sign) for positive results and – (minus sign) for negative results for the tested bioactive compounds.

## Results

### Bacterial isolation and characterization from wound samples

**Fig. 2:**
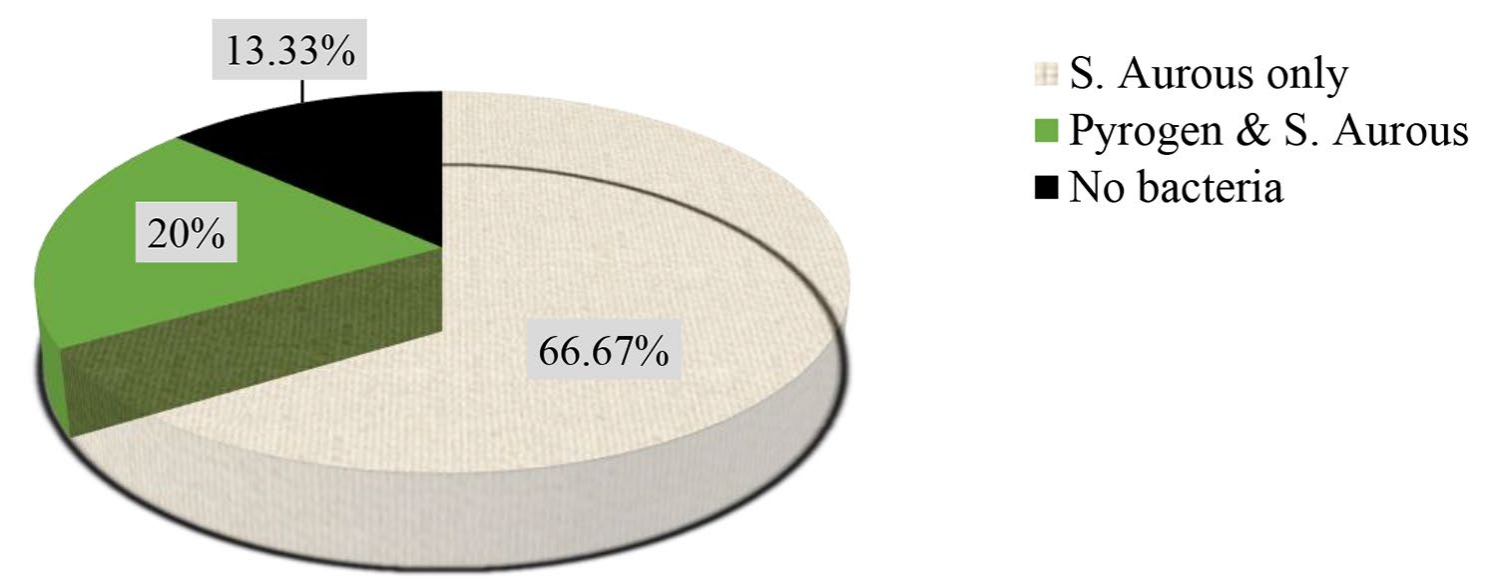
Type and percentage of identified bacteria among the total number of wound samples collected from patients with “*Shimetere”*

Overall, 15 wound samples of patients with *“Shimetere”* were collected from 5 patients, 3 (60%) were male, and their ages ranged from 15 to 40 years. Two types of bacterial strains were isolated from four (80%) of the patients with “Shimetere”. In 9 (60%) of the wound samples or 3 (60%) of the patients, only *S. aureus* was isolated, and in 3 (20%) wound samples or 1 (20%) of the patients, both *S. aureus* and *S. pyrogen* were identified. No bacteria were identified in the remaining 3 (20%) of the wound samples or 1 (20%) of the patients [Fig. 2].

### Antimicrobial susceptibility pattern of isolated bacteria

Both *S. aureus* and *S. pyrogen* bacteria were susceptible to reference antibiotics (cloxacillin and azithromycin). Cloxacillin (5 µg/disk) showed an average diameter in the IZ of 40.9 mm against isolated *S. aureus*. The average diameters in the IZ of azithromycin (15 µg/disk) were 41 and 46 mm against isolated *S. aureus* and *S. pyrogen* bacteria, respectively [Table 1].

**Table 1:** Antimicrobial susceptibility pattern of bacterial isolates from each wound sample

Standard drugs used	Patient serial number	IZ of bacterial isolated from each patient samples (diameter in mm)	
		Staphylococcus Aurous	Streptococcus Pyrogen
Azithromycin (15 µg/disk)	01	41.00	46.00
	02	42.00	-
	03	40.50	-
	04	40.50	-
	05	-	-
**Average IZ (Diameter (mm)** **±** **standard deviation)**		41.00 ± 0.7	46.00 ± 0.00
Cloxacillin (5 µg/disk)	01	43	x
	02	39	-
	03	42	-
	04	39.50	-
	05	-	-
**Average IZ (Diameter (mm)** **±** **standard deviation)**		40.9 ± 1.93	

Note: ^−^ Absence of isolated bacteria ^*x*^ Absence of the test

### Phytochemical screening of crude plant extracts

**Fig. 3:**
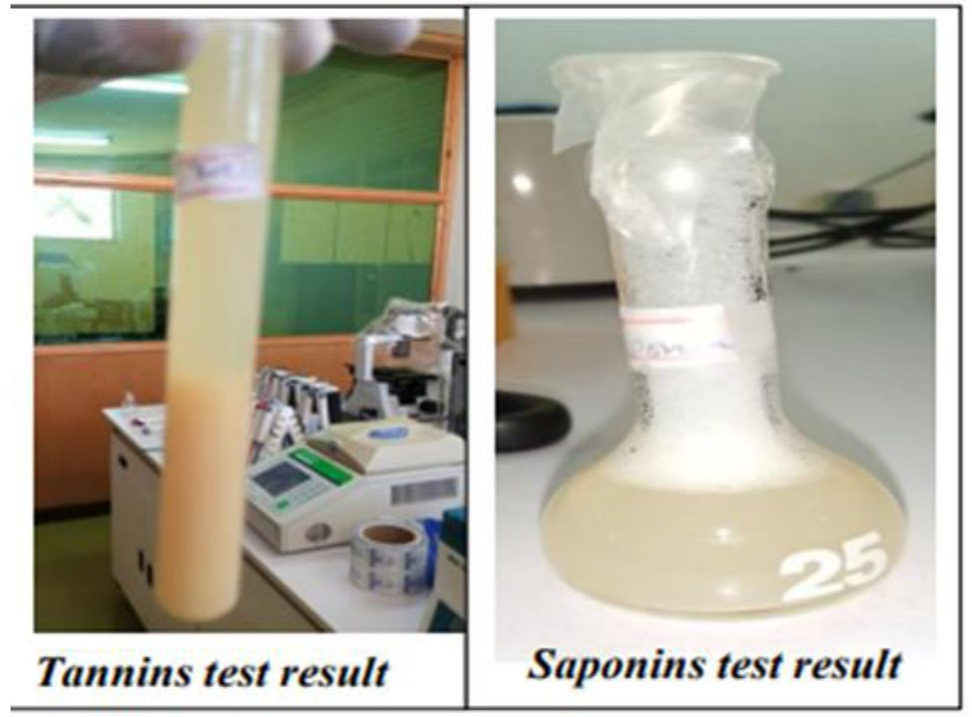
Moderately positive test results from phytochemical screening

The phytochemical screening of methanol extracts of *Polygala sadebeckiana Gürke* is summarized in Table 2; Fig. 3. The methanol extracts consisted of all tested secondary metabolites. Tannins and saponins were the most abundant secondary metabolites found in the crude extracts, while all other tested secondary metabolites were slightly available.

**Table 2:** Results of preliminary phytochemical screening of methanol extract of *Polygala sadebeckiana Gürke*

Test results	Secondary Metabolites						
	Saponin	Tannin	Polyphenol	Alkaloid	Flavonoid	Phytosterol	Glycoside
Abundant Present	+	+	-	-	-	-	-
Slightly present	-	-	+	+	+	+	+
Absent	-	-	-	-	-	-	-

Note: ^+^Yes, ^−^No

### Antibacterial activities of methanol crude extracts: *Agar-Well Diffusion Method*

**Fig. 4:**
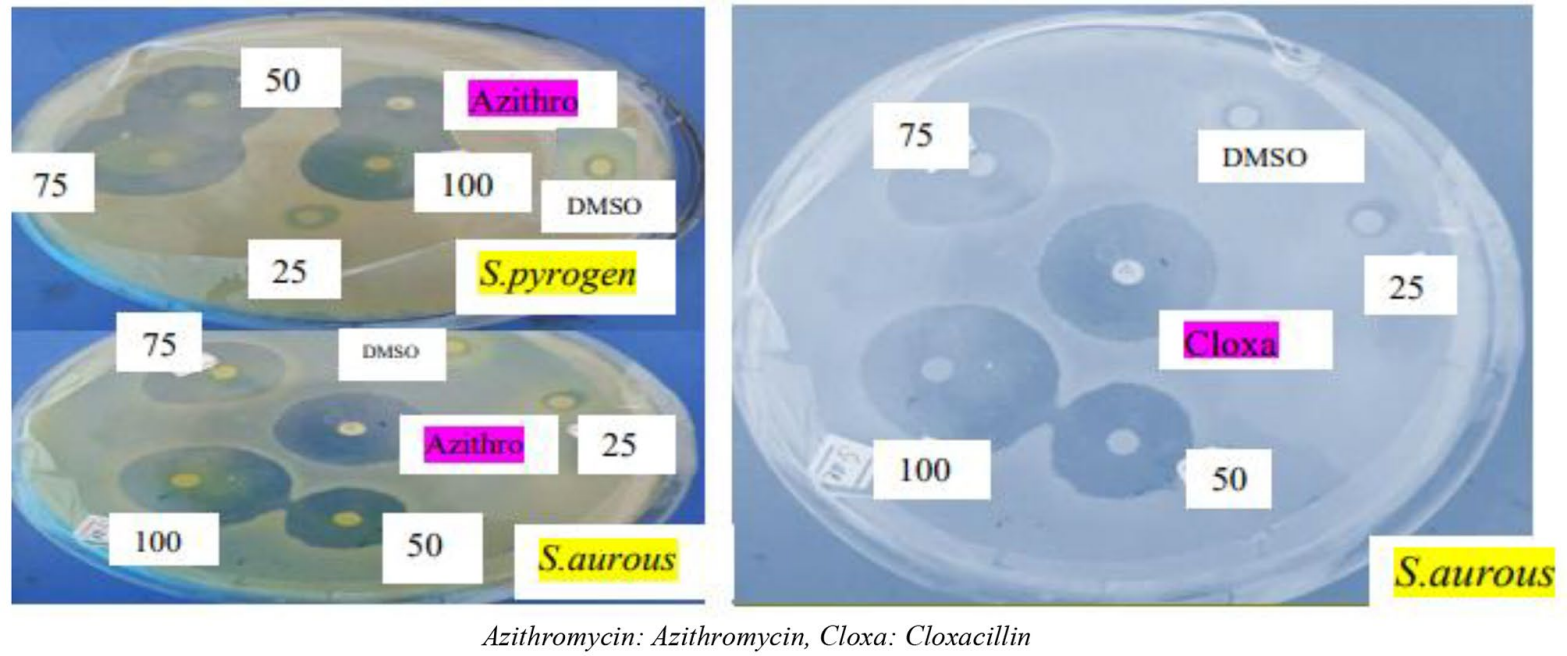
Inhibition zone of extracts with different concentrations against *S. aureus* and *S. pyrogen*

The results of the zone of inhibition (mm, diameter) generated by antibacterial activities of methanol crude extracts of *P. sadebeckiana* (Root) were measured and summarized in Table 3; Fig. 4. The crude extract concentrations were classified into 100, 75, 50 and 25 mg/mL in three replicates (3x) and exposed to *S. aureus* and *S. pyrogen*, which were isolated from patients with *“Shimetere”*.

**Table 3:** Antibacterial effects of methanol extracts of *Polygala sadebeckiana Gürke* at different concentrations (mean IZ of the three replicates (diameter in (mm) ± SD))

	[Concentration (mg/mL)]	Average IZ (Diameter (mm) ± standard deviation)			
		S. Aureus	*p* value	S. Pyrogen	*p* value
**Plant extracts**	**100 mg/mL**	27 ± 0.5	*0.000*	30.02 ± 0.42	*0.000*
	**75 mg/mL**	20.33 ± 0.58		24.50 ± 0.50	
	**50 mg/mL**	15.25 ± 0.66		19.07 ± 0.40	
	**25 mg/mL**	11.96 ± 0.48		15.77 ± 0.75	
**Azithromycin**	**15 µg/disk**	41 ± 0.7		46 ± 0.00	
**Cloxacillin**	**5 µg/disk**	40.9 ± 1.93		-	

### MIC and MBC: *broth dilution method*

MIC and MBC tests were carried out using a serial dilution of the crude extract by 5% DMSO. The methanol crude extracts with concentrations ranging from 0.5 µg/mL to 50 µg/mL were applied for both isolated bacterial strains (*S. aureus* and *S. pyrogen)*, and the results are shown in Table 4. The MIC and MBC values of the plant extracts were 1.67 and 10 mg/mL for *S. aureus* and 0.98 and 4 mg/mL for *S. pyrogen*, respectively. The MBC/MIC ratios of the plant extracts were 5.99 and 4.08 for *S. aureus* and *S. pyrogen* isolate, respectively.

**Table 4:** MIC and MBC of methanol extracts of *Polygala sadebeckiana Gürke* against identified bacterial strains (µg/mL)

Bacterial strains	The MIC and MBC of the Crude Extracts (mg/mL)		
	MIC	MBC	MBC/MIC
***S. Aureus***	1.67 ± 0.76	10.00 ± 1.00	5.99
***S. Pyrogen***	0.98 ± 1.32	4.00 ± 1.00	4.08

## Discussion

Infections of skin include pyoderma slight to severely necrotizing infections. It can be infected by several microorganisms, such as bacteria, parasites and fungi. Gram-positive bacteria such as *S.* aureus and Streptococcus species are the most common causes of skin infection [22]. Antibiotic therapy is recommended after performing an antibiotic susceptibility test [23].

The findings of our study showed that approximately 87% of wound samples were positive for *S. aureus* or *S. pyrogen* bacterial species, and *Staphylococcus aureus* was the most common (66.67%) isolate from the wound samples of *“Shimetere”* patients. The high prevalence might be because *S. aureus* is prevalent on the surface of skin and can easily enter wounds due to the disruption of the natural skin barrier. This is supported by previous studies in different parts of the world, such as Egypt [24], Nigeria [25, 26], Bangladesh [27], Indonesia [28], and Italy [29].

According to our findings, all isolated *S. aureus* were susceptible to cloxacillin, so all isolates were methicillin-susceptible *S. aureus* (MSSA). This finding is inconsistent with previous study findings performed in several parts of the world that reported the prevalence of MRSA bacterial isolates from skin and soft tissue infections. MRSA from skin infection in Dessie referral hospital of Northeast Ethiopia (28.3%) [30], Sohag university hospitals of Egypt (89%) [31] and Birendra military hospital of Nepal (53.09%) [32]. The possible reason for the absence of the MRSA strain in this study is that patients do not believe in modern medicine, so the bacteria inside could not be exposed to antibiotics to develop resistance. The small number of patients enrolled might also lessen the prevalence of MRSA strains.

There is a shift of focus to medicinal plants due to the increased antimicrobial resistance, severe adverse effects, high costs of synthetically available drugs and community perception towards modern health care practice [33]. Many studies have demonstrated that plant extracts are highly effective against bacterial microorganisms due to enormous kinds of secondary metabolites [33–35].

In this study, it was revealed that tannins and saponins were the most abundant secondary metabolites found, as the two phytochemical tests showed significantly visible positive results compared to other slightly available secondary metabolites in the methanol extracts of *Polygala sadebeckiana Gürke.* This result was slightly different from the previous study findings on the crude extracts of plants of the same family of *P. sadebeckiana* but different species. A study in Malaysia on *Polygala javana* plants revealed that polyphenols, alkaloids, tannins, phytosterols and flavonoids were dominantly present, but tannins were absent [36]. A similar study in Brazil on *Polygala boliviensis* showed a significant presence of alkaloids, saponins, flavonoids, phenols, tanins and steroids [37]. The differences in plant species, plant maturity during collection, soil conditions, fertilization, irrigation and pesticide use may be attributed to this disparity.

Plant extracts having chemicals with antimicrobial activity are usually alkaloids, flavonoids, polyphenols, saponins and tannins [38]. This study revealed that the antibacterial activity of crude extract against *S. aureus* and *S. pyrogen* was associated with the high concentration of tannins and saponins as secondary metabolites. This finding is supported by previous study results, such as the following: in Kenya, the presence of saponin and tannins showed greater activity among gram-positive bacteria than gram-negative bacteria [39]; in Korea, saponin extract had strong antibacterial activities against gram-positive bacteria by inhibiting the cell wall [40]; and in Nigeria, saponin extract exhibited an inhibitory effect on *S. aureus* but not on gram-negative organisms [41]. The mechanism of tannins and saponins might be the reason for their superior antibacterial activity against gram-positive bacterial strains.

For certain classes of antibiotics, the major killing effect against an organism is produced by either the time or the concentration of the drug at the binding site. In concentration-dependent bactericidal action, the rate of bactericidal activity depends on the magnitude of the maximum antibacterial concentration, whereas for drugs with time-dependent activity, the extent of bactericidal activity mainly depends on the duration of drug exposure at concentrations greater than the MIC [42].

The average IZs of the plant extracts with 100, 75, 50 and 25 mg/mL concentrations were 27, 20.33, 15.25, and 11.96 mm (*p* < *0.000*) for *S. aureus* and 30.02, 24.50, 19.07, and 15.77 mm (*p* < *0.000*) for *S. pyrogen* bacteria, respectively. This result showed that the increase in the concentration of *P. sadebeckiana* extract led to a significant increase in IZ diameter for both isolated bacterial strains. This finding was in agreement with previous studies on the antibacterial effects of medicinal plant extracts against similar bacterial species [38, 43–45]. However, the findings of the current study were different from those of other studies [19, 24, 43]. The possible reason may be that the antibacterial activity of the plant extract is concentration dependent.

The MIC is defined as the lowest concentration of antimicrobials that inhibited the visible growth of microorganisms after overnight incubation. MBC is the lowest concentration of antimicrobial that results in microbial death after subculturing the organism in antibiotic-free media [19].

The MIC and MBC values of the plant extracts were 1.67 and 10 mg/mL for *S. aureus* and 0.98 and 4 mg/mL for *S. pyrogen.* The results of the present study revealed that the methanol extract of *P. sadebeckiana* root was more potent against *S. pyrogen* than the *S. aureus* isolate. The plant extract in our study was relatively more potent in inhibiting the growth of *S. pyrogen* and/or *S. aureus* than several plant extracts in previous studies [19, 22, 35, 38, 46]. However, it was less potent than plant extracts in other study findings [35, 37]. The difference in potency of the plant extracts might be due to the diversity in susceptibility patterns of isolated *S. pyrogen* and *S. aureus* bacterial strains.

The MBC/MIC ratios of the methanol extracts were 5.99 and 4.08 against *S. aureus* and *S. pyrogen* isolates, respectively. An MBC/MIC ratio greater than 4 is usually considered to be a bacteriostatic effect, whereas values less than 4 show bactericidal effects [19]. Therefore, the methanol extracts of *P. sadebeckiana* were determined to be bacteriostatic for both bacterial isolates. The bacteriostatic nature of the plant extract might contribute to the recurrence of *“Shimetere”* because of reinfections despite the patient use roots of the plant as a traditional remedy.

## Conclusion

*S. aureus* and *S. pyrogen* were the two bacterial strains isolated from *“Shimetere”*, which were susceptible to the reference antibiotics. Tannins and saponins were the most abundant secondary metabolites found in the crude extract, although other tested secondary metabolites were slightly available. *Polygala sadebeckiana Gürke* extract had antibacterial activities against both isolated bacterial strains, and antibacterial activity was increased significantly by increasing the concentration of the plant extract. The potential mechanism of antibacterial action of the plant extract was cell wall synthesis inhibition.

## Recommendation

Further studies need to be conducted on *“Shimetere”* and other effects of *Polygala sadebeckiana Gürke* plant extracts.

### Limitation of the study

Bacteria were isolated from the wound samples of only five patients, which might decrease the possibility of identifying other bacterial species and resistant strains.

## Data Availability

The datasets analysed during this study are available from the corresponding author upon reasonable request.

## Abbreviations

DMSO: Dimethyl sulfoxide
MBC: Minimum Bactericidal Concentration
MDR: Multi-Drug Resistant
MIC: Minimum Inhibitory Concentration
MRSA: Methicillin-Resistant *Staphylococcus aureus*.
MSSA: Methicillin-Susceptible *Staphylococcus aureus*, IZ-Zone of Inhibition
